# Increased plasma membrane cholesterol in cystic fibrosis cells correlates with CFTR genotype and depends on *de novo *cholesterol synthesis

**DOI:** 10.1186/1465-9921-11-61

**Published:** 2010-05-20

**Authors:** Danjun Fang, Richard H West, Mary E Manson, Jennifer Ruddy, Dechen Jiang, Stephen F Previs, Nitin D Sonawane, James D Burgess, Thomas J Kelley

**Affiliations:** 1 Department of Chemistry, Case Western Reserve University, Cleveland, OH 44106, USA; 2 Department of Pediatrics and Pharmacology, Case Western Reserve University, Cleveland, OH 44106, USA; 3 Department of Nutrition, Case Western Reserve University, Cleveland, OH 44106, USA; 4 Department of Medicine, University of California, San Francisco, CA 94143, USA

## Abstract

**Background:**

Previous observations demonstrate that *Cftr*-null cells and tissues exhibit alterations in cholesterol processing including perinuclear cholesterol accumulation, increased *de novo *synthesis, and an increase in plasma membrane cholesterol accessibility compared to wild type controls. The hypothesis of this study is that membrane cholesterol accessibility correlates with CFTR genotype and is in part influenced by *de novo *cholesterol synthesis.

**Methods:**

Electrochemical detection of cholesterol at the plasma membrane is achieved with capillary microelectrodes with a modified platinum coil that accepts covalent attachment of cholesterol oxidase. Modified electrodes absent cholesterol oxidase serves as a baseline control. Cholesterol synthesis is determined by deuterium incorporation into lipids over time. Incorporation into cholesterol specifically is determined by mass spectrometry analysis. All mice used in the study are on a C57Bl/6 background and are between 6 and 8 weeks of age.

**Results:**

Membrane cholesterol measurements are elevated in both R117H and ΔF508 mouse nasal epithelium compared to age-matched sibling wt controls demonstrating a genotype correlation to membrane cholesterol detection. Expression of wt CFTR in CF epithelial cells reverts membrane cholesterol to WT levels further demonstrating the impact of CFTR on these processes. In wt epithelial cell, the addition of the CFTR inhibitors, Gly H101 or CFTR_inh_-172, for 24 h surprisingly results in an initial drop in membrane cholesterol measurement followed by a rebound at 72 h suggesting a feedback mechanism may be driving the increase in membrane cholesterol. *De novo *cholesterol synthesis contributes to membrane cholesterol accessibility.

**Conclusions:**

The data in this study suggest that CFTR influences cholesterol trafficking to the plasma membrane, which when depleted, leads to an increase in *de novo *cholesterol synthesis to restore membrane content.

## Background

Recent studies have identified alterations in cholesterol processing associated with cystic fibrosis (CF) [1-4]. The hypothesis of this study is that plasma membrane cholesterol accessibility as measured by electrochemical detection will correlate with CFTR genotype. Identifying this relationship between this cholesterol measurement and CFTR will help determine if this measurement can be potentially utilized as a biomarker of CF. With the development of new therapies targeting CFTR function, new methods of identifying efficacy are needed that are reliable and non-invasive. Mechanistically, it is proposed that rates of de novo cholesterol synthesis influence the membrane cholesterol measurement.

The cystic fibrosis transmembrane conductance regulator (CFTR) is a cAMP activated chloride channel of the ATP binding cassette (ABC) family [5,6]. A structurally similar protein within this family, ABCA1, is known to mediate cholesterol transport across the plasma membrane to high-density lipoprotein (HDL) [7,8]. The role of CFTR function in regulating cholesterol transport is unclear, particularly with respect to how plasma membrane cholesterol is regulated. It has been observed that cultured CF cells, as well as nasal and tracheal epithelium from CFTR null mice, exhibit a significant increase in plasma membrane cholesterol accessibility [3]. Although structurally similar to ABCA1, there is no evidence that CFTR is capable of directly modulating cholesterol movement. We have previously demonstrated that *de novo *cholesterol synthesis is elevated in the lungs of *Cftr *-/- mice [3]. Evidence does suggest that membrane cholesterol content can be regulated by *de novo *synthesis [9,10]. Koter et al demonstrate that statin treatment reduces membrane cholesterol content in erythrocytes from 2.24 +/- 1.69 to 1.17 +/- 0.75 mg cholesterol/mg protein, a 47% reduction [9]. It is possible that increased membrane cholesterol in CF plasma membrane is related to increased *de novo *cholesterol synthesis.

Regardless of whether cholesterol processing changes observed in CF cells and tissues are directly involved in the pathology of CF, these cholesterol changes are potentially good, accessible indirect markers of CF-related cell signaling. The goal of this study is to determine if CFTR genotype correlates with plasma membrane cholesterol detection and to determine if *de novo *cholesterol synthesis contributes to this measure. Data demonstrate a clear CFTR genotype correlation with ΔF508 CFTR mice exhibiting higher membrane cholesterol content and increased *de novo *cholesterol synthesis relative to R117H CFTR mice. Other studies demonstrate the relationship between membrane cholesterol and CFTR by examining restoration of wt CFTR in CF epithelial cells and examining the impact of acute CFTR inhibition.

## Methods

### Cell culture

Human epithelium 9/HTEo-cells over expressing the CFTR R domain (pCEPR) and mock-transfected 9/HTEo-cells (pCEP), the wild type phenotype, were a generous gift from the lab of Dr. Pamela B. Davis (Case Western Reserve University). Cells were cared for as previously described [11]. IB3-1 cells, human epithelial with the delta F508 mutation (CF-phenotype), and S9 cells, IB3-1 cell stably transfected with the full-length wt CFTR (control) were a generous gift from Pamela L. Zeitlin (Johns Hopkins University, Baltimore, MD). These cells were grown at 37°C in 95% O_2_-5% CO_2 _on Falcon 10 cm diameter tissue culture dishes in LHC-8 Basal Medium (Biofluids Camarillo, CA) with 5% FBS.

### Mice

Mice homozygous for the ΔF508 CFTR mutation were described previously [12], as were mice carrying the R117H CFTR mutation [13]. Mice heterozygous for CFTR expression (*Cftr*_*tm1Unc*_) were obtained from Jackson Laboratories (Bar Harbor, MA). All mice were provided by the CF Center animal core facility at Case Western Reserve University. CFTR wild-type mice were siblings of *Cftr *+/- mice. All mice were used between six and eight weeks of age. All mice were used between six and eight weeks of age and are back-crossed over ten generations onto a C57Bl/6 background. Mice were cared for in accordance with the Case Western Reserve University IACUC guidelines by the CF Animal Core Facility.

### Electrochemical measurements of cholesterol

Platinum microelectrodes are fabricated in house (4 μm diamter wire for cell work and 100 μm diameter wire for tissue measurements, Goodfellow Corp.) as described [14,15]. Platinum wire is inserted into glass capillaries (Kimax-51, Kimble products) and placed inside a heated platinum coil. The glass is pulled to create a thin insulating layer on the platinum wire. The capillary microelectrodes are polished using a beveling machine (WPI, Inc.) to produce a disk electrode. The microelectrodes are immediately immersed in a 5 mM hexane solution of 11-mercaptoundecanoic acid (95%, Aldrich Chem. Co) for 2 h to form a carboxylic acid terminated monolayer on the electrode surface. Then, the microelectrodes are treated 2 mM 1-ethyl-3-(3-dimethylaminopropyl) carbodiimide (EDC) (Sigma Chem. Co.) in 100 mM PBS solution (pH 7.4) for 30 min. to activate the carboxyl groups to an acylisourea intermediate. The modified electrode is immersed in 1 mg/ml recombinant cholesterol oxidase (WAKO Chemical USA, Inc., 42.0 units/mg) solution for 3 hrs allowing this intermediate to react with amine immobilizing the enzyme on the electrode surface.

### Data Acquisitions

Amperometric measurements are conducted using a two-electrode cell and a voltammeter-amperometer (Chem-Clamp, Dagan corp.). The three-pole Bessel filter in the voltammeter-amperometer is set to 100 Hz. The output is further processed using a noise-rejecting voltmeter (model 7310 DSP, Signal Recovery Inc.) to digitally filter 60-Hz noise. An Ag/AgCl (1 molar KCl) reference electrode is used for all experiments, and the applied potential is 780 mV versus NHE for all experiments. All experiments are performed in 100 mM phosphate buffer (pH 7.4) at 36°C. Single cells and excised tissue are captured by a capillary prepared in house using an IM-6 microinjector (Narishige International USA, Inc.). The electrode is initially positioned about 50 μm from the cell surface or tissue inner edge for acquisition of baseline data. The electrode is repositioned for contacting the biological sample and acquisition of electrode response.

### Measuring cholesterol synthesis in vivo

*CFTR*_*tm1Unc *_mice and the matched controls were given an intraperitoneal injection (i.p.) (~ 24 μl per g body weight) of deuterated saline (9 g NaCl in 1000 ml of 99% 2H2O, Sigma-Aldrich, St. Louis, MO). After 8 h, mice were sacrificed using carbon dioxide. Blood was taken from the heart and plasma collected. Whole lungs were collected. Tissue samples were hydrolyzed in 1N KOH/70% ethanol (v/v) for 2 at 70°C vortexing occasionally. Samples were then evaporated to dryness, redissolved in 2 ml of water and acidified using 12N HCl. Cholesterol was extracted twice by addition of ethyl ether (3 ml). The pooled ether extracts were evaporated to dryness under nitrogen and then converted to the trimethylsilyl cholesterol derivatives by reacting with 60 μl of bis(trimethylsilyl) trifluoroacetamide + 1% trimethylchlorosilane (Regis, Morton Grove, IL) (TMS) at 60°C for 20 min. The 2H-labeling of cholesterol was determined using an Agilent 5973N-MSD equipped with an Agilent 6890 GC system. The cholesterol was run on a DB17-MS capillary column (30 m × 0.25 mm × 0.25 μm). The oven temperature was initially held for 1 min at 150°C, then increased by 20°C per min to 310°C and maintained for 8 min. The split ratio was 20:1 with helium flow 1 ml per min. The inlet temperature was set at 270°C and MS transfer line was set at 310°C. Under these conditions, cholesterol elutes at ~11.1 min. Electron impact ionization was used in all analyses with selected ion monitoring of m/z 368-372 (M0-M4, cholesterol), dwell time of 10 ms per ion.

The ^2^H-labeling of mice plasma water was determined by exchange with acetone. Briefly, plasma was diluted 2-fold with distilled water and reacted with 2 μl of 10 N NaOH and 4 μl of a 5% (v/v) solution of acetone in acetonitrile for 24 h. Acetone was extracted by addition of 600 μl of chloroform followed by addition of 0.5 g Na_2_SO_4_. Samples were vigorously mixed and a small aliquot of the chloroform was transferred to a GC-MS vial. Acetone was analyzed using Agilent equipment described above. The oven temperature program was: 60°C initial, increase by 20°C per min to 100°C, increase by 50°C per min to 220°C and maintain for 1 min. The split ratio was 40:1 with a helium flow of 1 ml per min. The inlet temperature was set at 230°C and the mass spectrometer transfer line was set at 245°C. Acetone eluted at ~1.5 min. The mass spectrometer was operated in the electron impact mode (70 eV). Selective ion monitoring of m/z 58 and 59 was performed using a dwell time of 10 ms per ion.

### Calculation of cholesterol synthesis

Protocols followed in [3] were used. Briefly, rates of de novo cholesterol synthesis were calculated using the formula: Total labeling ([(M_1 _× 1) + (M_2 _× 2) + (M_3 _× 3) + (M_4 _× 4)])/n/^2^H-labeling of plasma water × time where M*i *represents isotope labeled isomeric species of cholesterol (M_1 _being singly labeled, M_2 _doubly labeled) and "n" represents the number of exchangeable hydrogens, assumed to be 25 for cholesterol.

### Synthesis of Inactive-CFTR_inh_-172: 5-(N,N-dimethylphenyl)methylene)-2-thioxo-3-[3-(trifluoromethyl)phenyl]-4-thiazolidinone

Mixture of 2-thioxo-3-(3-trifluoromethyl phenyl)-4-thiazolidinone (110 mg, 0.4 mM, synthesized according to Sonawane et al., [16], 4-(*N,N*-dimethyl)benzaldehyde (59 mg, 0.4 mM), and sodium acetate (50 mg) in glacial acetic acid (1 ml) was refluxed for 4 h. Solvent was evaporated, residue dissolved in ethyl acetate, filtered, and silica gel (1 g) was added. Compound on silica gel was purified by normal phase flash chromatography to yield 68 mg yellow-orange crystals (yield 42%); mp: 224-226 °C; MS (ES+) (*m/z*): [M+H]_+ _calculated for C_19_H_15_F_3_N_2_OS_2_, 409.4, found 409.3.

## Results

### Membrane cholesterol genotype comparison

**Figure 1:**
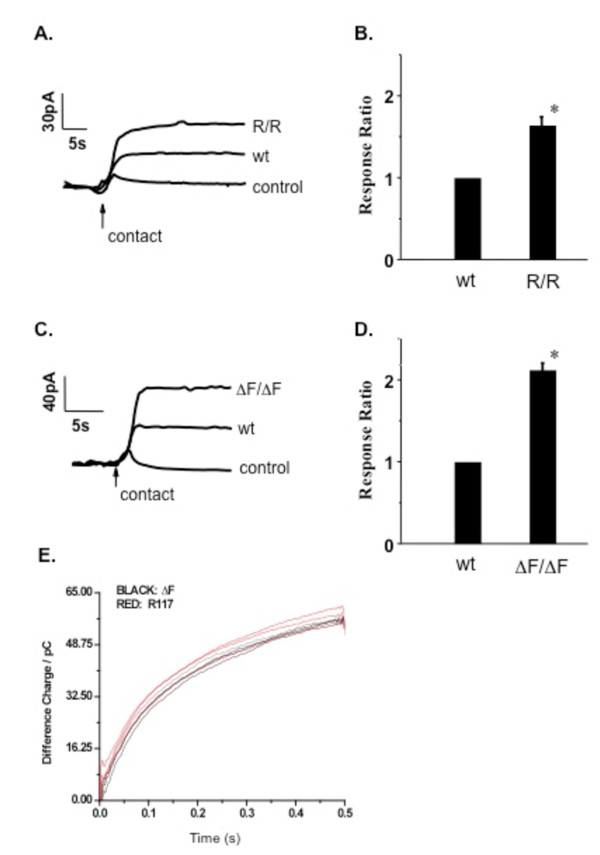
**Electrochemical determination of membrane cholesterol content from mouse nasal epithelium**. A, C) Representative traces of membrane cholesterol determination in excised nasal epithelium from *Cftr R117H/R117H *(R/R) mice and *Cftr *ΔF508/ΔF508 (ΔF/ΔF) mice, respectively, compared to sibling *Cftr +/+ *(wt) mice. B, D) Quantification of responses between *Cftr R/R *and sibling *Cftr +/+ *(wt) nasal tissue and *Cftr *ΔF/ΔF nasal tissue compared to *Cftr *+/+ (wt) siblings. Responses are reported relative to wt response (response ratio) to indicate the fold-increase in response. Error bars represent SEM, n = 4 for each. Significance determined by *t *test. *p < 0.001. E) Representative traces of wt mice from the ΔF508 (ΔF) and R117H colonies. Mean response for wt mice in the ΔF and R117H colonies are 54.5 +/- 0.5 pC and 55.7 +/- 1.5 pC, respectively.

Previous work has demonstrated that cultured cell models of CF and nasal and tracheal epithelium from *Cftr *-/- mice exhibit an increase in membrane cholesterol content [3,17]. In order to determine if membrane cholesterol measurement correlates with *Cftr *genotype, membrane cholesterol was measured in nasal epithelium isolated from mice homozygous for either the R117H (R/R) or the ΔF508 (ΔF/ΔF) CFTR mutations. Membrane cholesterol content as measured by response ratio is elevated in both R/R and ΔF/ΔF nasal epithelium (1.64 +/- 0.09 (R/R), p < 0.001; 2.14 +/- 0.10 (ΔF/ΔF), p = 0.01) compared to age-matched sibling wt controls (Figure 1). These data demonstrate that either a mild or severe disease related CFTR mutation will result in an increase in membrane cholesterol, with a larger magnitude increase in the ΔF/ΔF tissue. The magnitude increase in membrane cholesterol in ΔF/ΔF mouse nasal tissue is identical to what is observed in *Cftr *-/- nasal tissue [3]. This measurement does not determine absolute cholesterol content, only cholesterol accessible to the electrode at the outer leaflet of the plasma membrane. This increased accessibility could be due to increased total content, cholesterol displacement from the lipid bilayer, or efflux. These data demonstrate that membrane cholesterol measurements can differentiate between genotypes. WT mice from the ΔF508 colony and the R117H colony were directly compared and no difference in membrane cholesterol measurement was observed.

### Effect of CFTR correction on membrane cholesterol content

**Figure 2:**
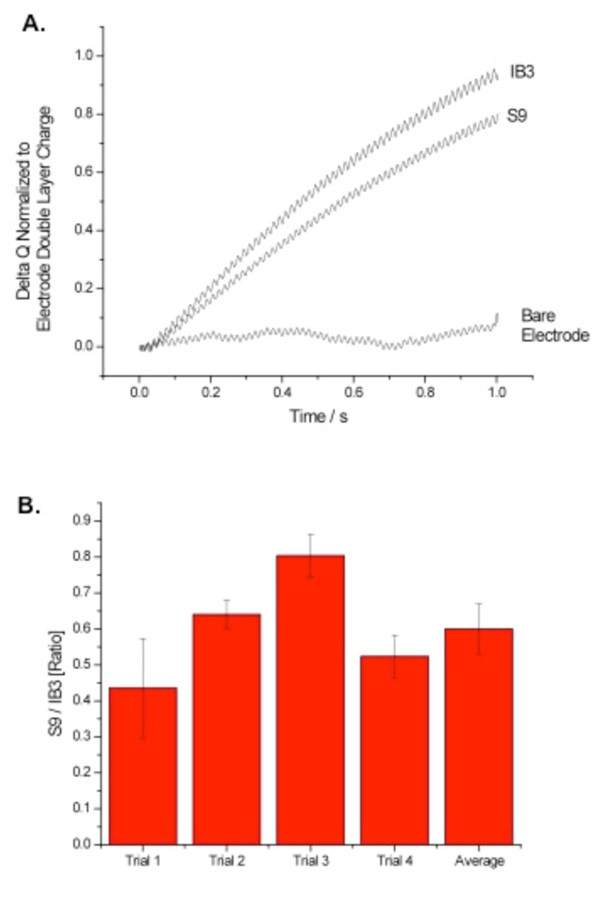
**Electrochemical determination of membrane cholesterol content in CFTR corrected CF cells**. A) Representative traces of membrane cholesterol determination in IB3 CF cells and in CFTR-corrected IB3 cells (S9). B) Quantification of responses between *IB3 and S9 cells*. Responses are reported relative to wt response (response ratio) to indicate the fold-increase in response. Error bars represent SEM, n = 4 for each. Significance determined by *t *test. *p < 0.001.

The above data demonstrate that mutation of CFTR results in an increase in membrane cholesterol content. To verify if this effect is truly dependent on CFTR, membrane cholesterol accessibility in the CF epithelial cell line IB3 cells (ΔF508/W128X) and in S9 cells (IB3 cells stably expressing wt CFTR) was measured. The goal of this experiment is to determine if restoring wt CFTR expression in a CF cell will restore normal membrane cholesterol homeostasis. As shown in Figure 2, S9 cell membrane cholesterol content is significantly reduced compared to parent IB3 cells. These data support the hypothesis that wt CFTR is required to maintain membrane cholesterol homeostasis.

### The effect of pharmacological inhibition of CFTR on membrane cholesterol content

**Figure 3:**
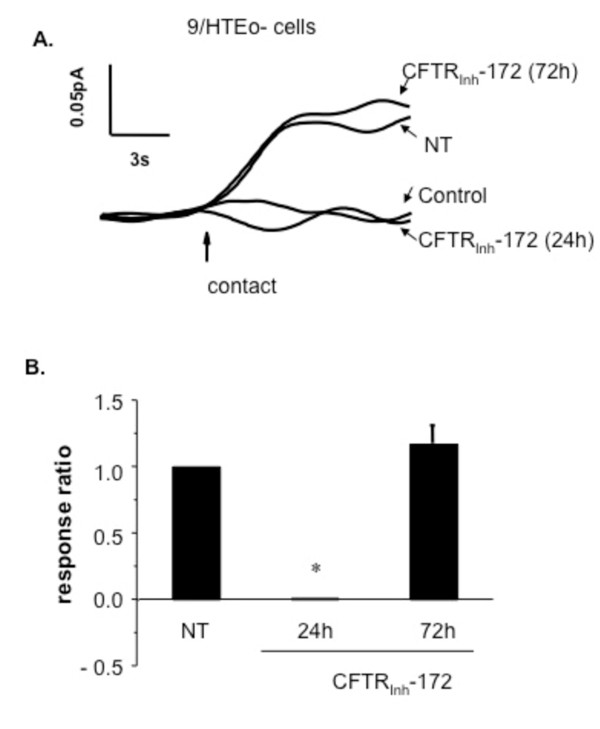
**Effect of 24 h CFTR inhibition and 72 h CFTR inhibition with CFTR_inh_-172 (20 μM) on membrane cholesterol content**. A) Representative traces of membrane cholesterol determination in 9/HTEo-cells after treatment with the CFTR inhibitor CFTR_inh_-172 for either 24 h or 72 h with fresh inhibitor placed on cells every 24 h or cells with no treatment (NT). B) Quantification of responses between cells with no treatment (NT) and cells with acute (24 h) or chronic (72 h) CFTR inhibition. Responses are reported relative to NT response (response ratio) to indicate the fold difference in response. Error bars represent SEM, n = 4 for each. Significance determined by ANOVA relative to NT samples. *p < 0.001.

Data indicate a clear influence of CFTR genotype on membrane cholesterol content regulation. The mouse models and cell models used model chronic alterations to CFTR. The goal of this experiment is to determine the impact of acutely modulating CFTR with pharmacological inhibitors on membrane cholesterol. 9/HTEo-epithelial cells were treated with the CFTR selective inhibitor CFTR_inh_-172 (20 μM) for 24 h and membrane cholesterol content measured electrochemically [16,18]. CFTR inhibition under these conditions with this compound has been reported to reproduce cell regulation profiles associated with CF inflammation [19]. Contrary to cellular and *in vivo *CF models, acute CFTR inhibition resulted in a dramatic reduction in membrane cholesterol accessibility (Figure 3). This finding suggested that increased membrane cholesterol content in CF is actually a secondary response. To test this hypothesis, 9/HTEo-cells were exposed to CFTR_inh_-172 (20 μM) continuously for 72 h being replenished with fresh drug every 24 h. Longer CFTR inhibition results in a rebounding of membrane cholesterol that begins to exceed baseline levels, although not statistically significant at this time point (Figure 3). These data suggest that alterations in cholesterol processing in CF may be due to feedback mechanisms triggered by initially diminished membrane cholesterol content in response to lost CFTR function. However, membrane cholesterol does not significantly exceed baseline levels after 72 h. Pharmacological inhibition of CFTR with CFTR_inh_-172 does not recapitulate the whole process of CF alterations in cholesterol processing and the source of increased membrane cholesterol content still needs to be determined.

**Figure 4:**
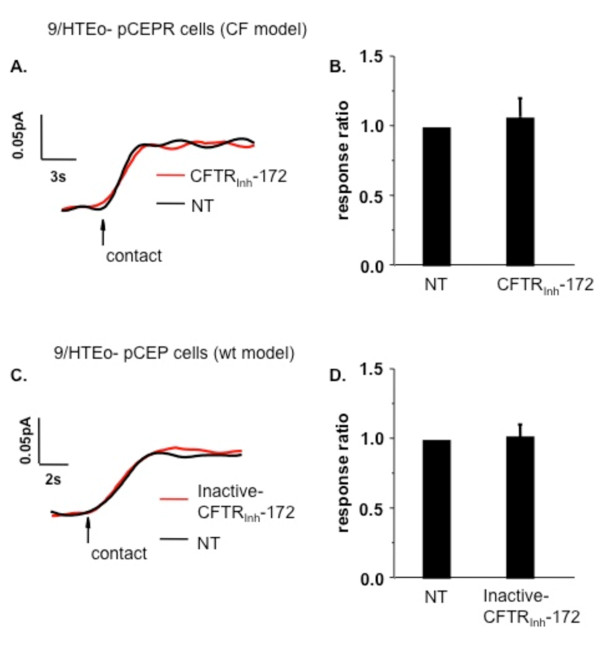
**Specificity of CFTR inhibition in regulating membrane cholesterol content**. A) Representative traces of membrane cholesterol determination in 9/HTEo-pCEPR (CF) cells after treatment with the CFTR inhibitor CFTR_inh_-172 for 24 h (red line) or cells with no treatment (NT, black line). B) Quantification of responses between pCEPR (CF) cells with no treatment (NT) and cells with acute (24 h) addition of the CFTR inhibitor CFTR_inh_-172 (20 μM). Responses are reported relative to NT response (response ratio) to indicate the fold-difference in response. Error bars represent SEM, n = 4 for each. Significance determined by *t *test. No significant difference was found. C) Representative traces of membrane cholesterol determination in 9/HTEo-pCEP (wt) cells after treatment with Inactive-CFTR_inh_-172 for 24 h (red line) or cells with no treatment (NT, black line). D) Quantification of responses between pCEP (wt) cells with no treatment (NT) and cells with acute (24 h) addition of the Inactive-CFTR_inh_-172 (20 μM). Responses are reported relative to NT response (response ratio) to indicate the fold-difference in response. Error bars represent SEM, n = 4 for each. Significance determined by *t *test. No significant difference was found.

To assure that CFTR_inh_-172 was likely mediating the drop in membrane cholesterol via CFTR inhibition, two controls were performed. First, the influence of CFTR_inh_-172 on CF-model pCEPR 9/HTEo-cells was examined. The pCEPR cells are lacking CFTR function due to the over expression of the regulatory (R) domain and have been shown to exhibit the phenotype of increased membrane cholesterol content compared to wt controls [3,11]. If the initial drop in membrane cholesterol content is due to CFTR inhibition, CFTR_inh_-172 should have no effect in pCEPR cells. Exposure of CF-model pCEPR cells to CFTR_inh_-172 (20 μM) for 24 h indeed has no impact on membrane cholesterol content (Figure 4A, B). These data support the finding that the initial drop in membrane cholesterol is due to acute CFTR inhibition, and further suggest that the subsequent increase in membrane cholesterol content in CF cells is due to a secondary feedback response. A second control consisted of treating 9/HTEo-cells with an inactive form of CFTR_inh_-172 (Inactive-CFTR_inh_-172) to verify that some nonspecific drug interaction was not responsible for the decrease in membrane cholesterol. As shown in Figures 4C and 4D, Inactive-CFTR_inh_-172 had no influence on membrane cholesterol content. These data strongly support the findings that acute inhibition of CFTR function leads to decreased membrane cholesterol content.

**Figure 5:**
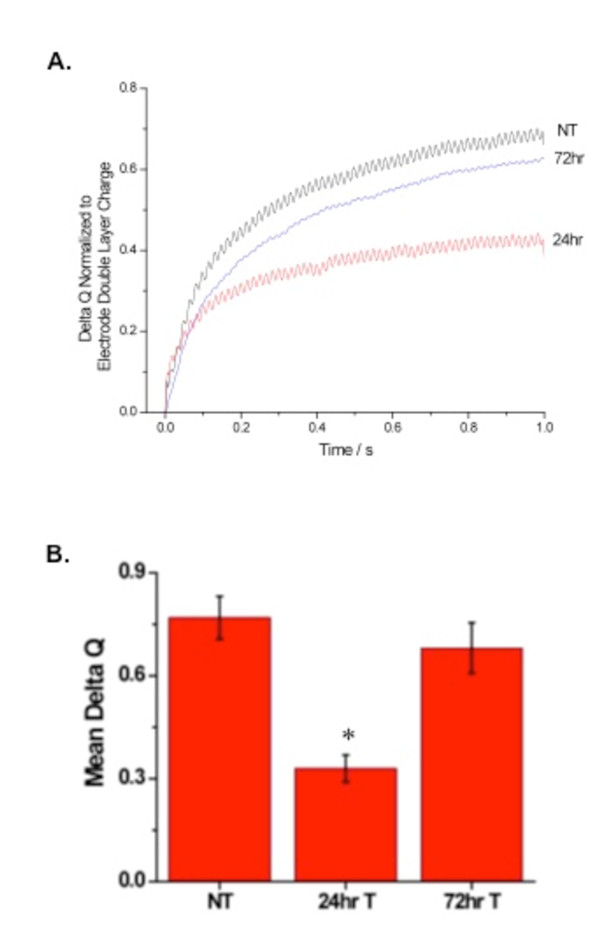
**Effect of 24 h CFTR inhibition and 72 h CFTR inhibition with Gly H101 (20 μM) on membrane cholesterol content**. A) Representative traces of membrane cholesterol determination in 9/HTEo-cells after treatment with the CFTR inhibitor Gly H101 for either 24 h or 72 h with fresh inhibitor placed on cells every 24 h or cells with no treatment (NT). B) Quantification of responses between cells with no treatment (NT) and cells with acute (24 h) or chronic (72 h) CFTR inhibition. Responses are reported relative to NT response (response ratio) to indicate the fold-difference in response. Error bars represent SEM, n = 4 for each. Significance determined by ANOVA relative to NT samples. *p < 0.001.

To confirm that inhibition of CFTR influences membrane cholesterol content, the effect of a second pharmacological inhibitor of CFTR, Gly H101 (10 μM), was examined. A similar, but slightly modified, electrochemical technique was used to test the influence of Gly H101 on membrane cholesterol. A background subtraction analog chronocoulometry method is used to quantify hydrogen peroxide production, which correlates to cholesterol content. With the electrode held in contact with the cell surface, several measurements reflecting membrane cholesterol are collected. Consistent with CFTR_inh_-172 results, cells treated with Gly H101 for 24 h demonstrate a significant decrease in cholesterol content that rebounds to baseline levels by 72 h (Figure 5).

### Heterozygote effect

**Figure 6:**
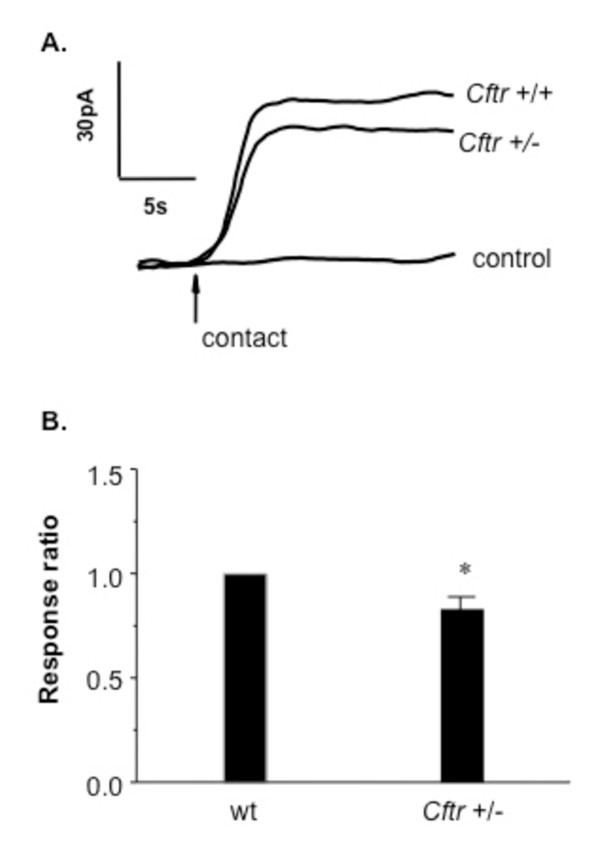
**Comparison of nasal epithelium membrane cholesterol content in *Cftr *+/+ and *Cftr *+/- mice**. A) Representative traces of membrane cholesterol determination in excised nasal epithelium from *Cftr +/+ *(wt) mice and *Cftr +/- *mice. B) Quantification of electrochemical membrane cholesterol determination between *Cftr +/+ *(wt) nasal tissue and *Cftr *+/- nasal tissue. Responses are reported relative to wt response (response ratio) to indicate the fold-difference in response. Error bars represent SEM, n = 4 for each. Significance determined by *t *test. *p = 0.008.

The finding that acute CFTR inhibition leads to significant membrane cholesterol depletion, coupled with the observations that both mild and severe CFTR mutations result in elevated membrane cholesterol content, prompted the examination of what the effect of CFTR heterozygosity woud be on membrane cholesterol accessibility. The goal of the study was to determine if there is a CFTR dose effect with *Cftr *+/- mice having elevated membrane cholesterol intermediated between wt and CF models, or if there was actually a loss of membrane cholesterol as seen with acute CFTR inhibition. As shown in Figure 6, *Cftr *+/- nasal epithelium exhibits a relatively slight, but significant loss of membrane cholesterol (0.87 +/- 0.04 fold compared to wt, p < 0.01). These data suggest that CFTR heterozygosity impacts cholesterol movement to the plasma membrane. These data can be consistent with the pharmacological inhibition of CFTR data in that CFTR may be involved in cholesterol movement to the membrane and reduced membrane cholesterol triggers a feedback response. The slight drop in membrane cholesterol content in *Cftr *+/- nasal tissue, although statistically significant, is likely physiologically insufficient to trigger a compensatory mechanism to increase cholesterol synthesis.

### Increased lung cholesterol synthesis in two different mouse models of CF

**Figure 7:**
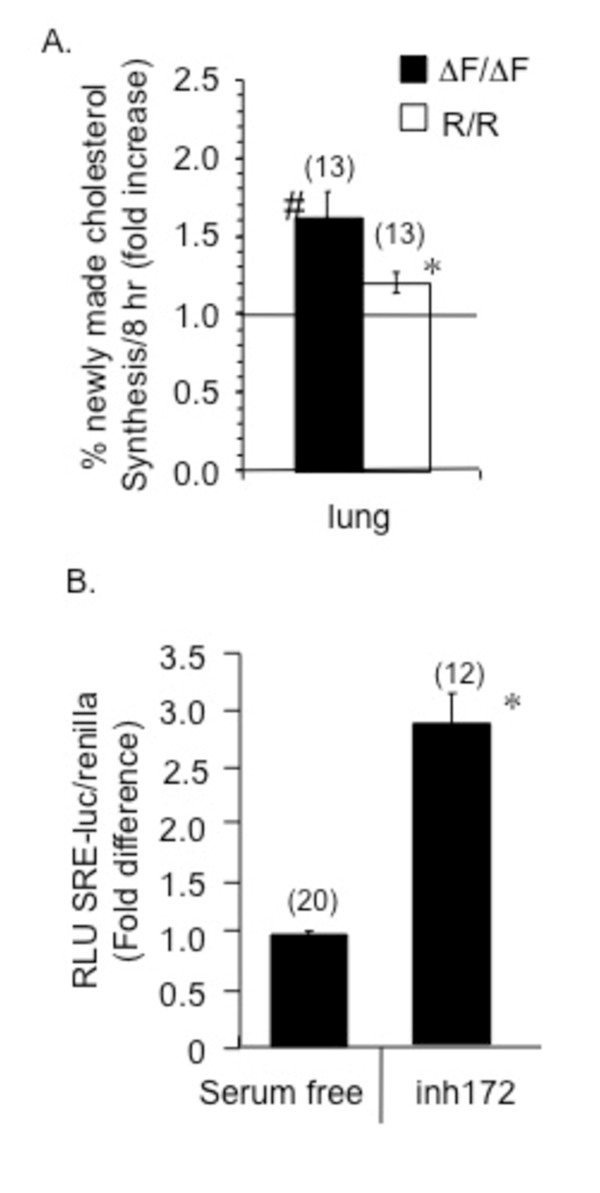
***De novo *cholesterol synthesis in CF mouse lung compared to matched controls**. A) Deuterium incorporation into newly synthesized tissue cholesterol was measured by GC/MS. Data is normalized to each tissue wt control (represented by dotted line) and reported as fold increase of % newly synthesized cholesterol/8 h. Filled bars represent ΔF/ΔF mice and open bars represent R/R mice. The number of replicates is shown in parenthesis above each bar and represents individual assay on multiple tissue samples obtained over 3 experiments. *p < 0.05, #p < 0.01. B) Increased SRE response in CFTR_inh_-172 treated control epithelial cells. 9/HTEo-pCEP (wt) cells were incubated in serum free conditions for 24 h with or without 20 μM CFTR_inh_-172 (INH-172) in serum free media for an additional 24 h. 9/HTEo-pCEP are open bars. Data are normalized to serum free NT control levels over 3 experiments. Number (n) of samples is in parenthesis above each bar. Significance was determined by *t *test. Error bars represent SEM. * p < 0.0001.

Lange and Steck described a relationship between membrane cholesterol content and the regulation of cholesterol synthesis [20]. A potential mechanism for the rebound of membrane cholesterol observed with pharmacological CFTR inhibitors would be an increase in *de novo *synthesis in response to transient decreases in membrane cholesterol content in response to dysfunctional CFTR. A prediction based on this mechanism would be that cholesterol synthesis should correlate with CFTR genotype in relation to membrane cholesterol content in the airways. To test this prediction, lung *de novo *cholesterol synthesis in ΔF/ΔF and R/R mice was measured. Deuterium incorporation into cholesterol of specific tissue was determined by GC/MS analysis. Results reveal a 1.6 + 0.2 fold (p = 0.009) increase in % new cholesterol synthesis in the lung of ΔF/ΔF mouse lung compared to controls and a 1.2 + 0.1 fold (p = 0.04) in the lungs of R/R mice compared to respective controls (Figure 7A). Increased cholesterol synthesis in the lungs of two other CF mouse models validates our previous findings in *Cftr-/- *mouse tissue [3], and a more severe CFTR mutation correlates with greater increases in the rate of cholesterol synthesis in the lung, supporting the importance of CFTR function in influencing cholesterol synthesis.

### Increased sterol response element (SRE) activation in response to CFTR inhibition

The above data suggest that CFTR genotype influences the regulation of *de novo *cholesterol regulation. Based on the above results it is predicted that treatment with CFTR inhibitors should result in SRE activation. To test this prediction, the effect of the CFTR inhibitor CFTR_inh_-172 on SRE activation was examined utilizing an SRE-luciferase construct. The construct contains SRE binding sites of the promoter region of HMG-CoA synthase, the rate-limiting enzyme regulating *de novo *cholesterol synthesis. 9/HTEo-cells were treated with 20!M CFTR_inh_-172 for 24 hours and assayed for SRE responsiveness. Control epithelial cells treated with CFTR_inh_-172 showed a significant increase of 2.8 + 0.3 fold (p < 0.0001) above control levels (Figure 7B). These data are consistent with the *in vivo *data above that loss of CFTR function leads to an increase in *de novo *cholesterol synthesis.

### Regulation of membrane cholesterol content by cholesterol synthesis in CF

**Figure 8:**
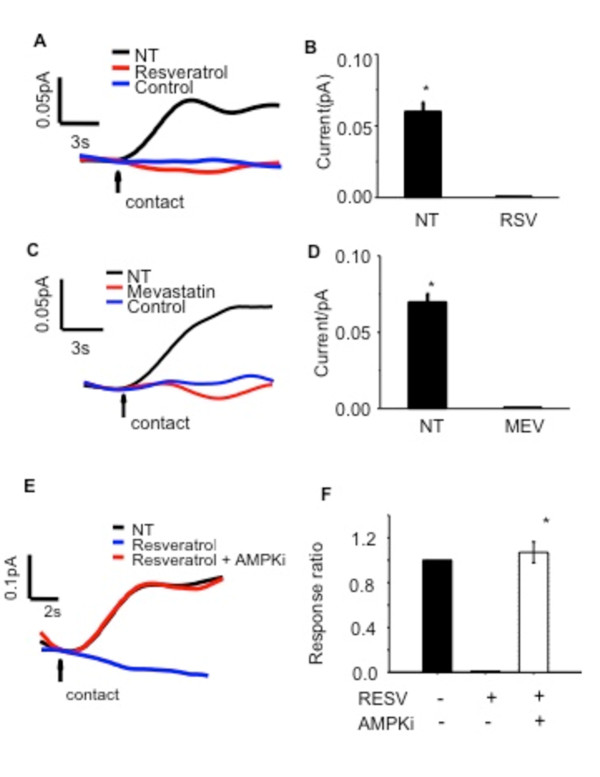
**Role of cholesterol synthesis in modulating membrane cholesterol content**. (A) Membrane cholesterol content, measured as current (pA, picoamps), is decreased in resveratrol (RSV) treated 9HTEo-pCEPR (CF) epithelial cells. Control experiment is done using bare Pt on untreated cells. (B) Resveratrol treated CF epithelial cells show decreased membrane cholesterol content (0.06 pA +/- 0.01 *p-value <0.001) compared to untreated cells. Data are shown as mean of four separate experiments. (C and D) Mevastatin (MEV) treated 9HTEo-pCEPR (CF) epithelial cells show a similar significant decrease in membrane cholesterol content (0.07 pA +/- 0.01 *p-value <0.001). Data are shown as mean of four separate experiments. Error bars are SEM in all experiments. (E) Resveratrol's ability to decrease membrane cholesterol content is blocked in the presence of the AMPKi. Current measured as picoamps (pA). (F) AMPKi increases membrane cholesterol content above baseline compared to resveratrol treated cells (1.07 +/- 0.09, *p < 0.001). Untreated CF cells normalized to one. Data are from three separate experiments. Error bar is SEM.

The above data suggest a relationship between cholesterol synthesis and membrane cholesterol content. To test directly whether cholesterol synthesis impacts membrane cholesterol content in CF cells and tissues, cholesterol synthesis was inhibited with two unrelated compounds, mevastatin and resveratrol, and the impact on membrane cholesterol content examined. Only CF cells were tested due to increased cholesterol signal at the membrane with electrochemical detection. It is anticipated that the relationship between cholesterol synthesis and membrane cholesterol accessibility would be the same in wt cells, but this relationship was not tested directly. Resveratrol was used because it is unrelated to the statin compounds and Do et al have shown that resveratrol reduces cholesterol synthesis *in vivo *in apo E-deficient mice through an AMP kinase (AMPK)-dependent mechanism [21]. In order to test the hypothesis that cholesterol synthesis contributes to membrane cholesterol content in CF cells, the impact of mevastatin and resveratrol on membrane cholesterol was examined. CF model 9/HTEo-pCEPR cells were chosen since they are cultured cells that exhibit the increased membrane cholesterol that is observed *in vivo *in mouse models of CF [6]. Cells were treated with either mevastatin (50 μM) or resveratrol (50 μM) for 24 hours. Resveratrol treated CF epithelial cell membrane cholesterol content is significantly decreased (0.06 pA +/- 0.01, p-value <0.001) compared to untreated cells (Figure 8A, B). The control trace represents a measurement taken with a bare electrode not modified with cholesterol oxidase to confirm the cholesterol sensitivity of the measurement. CF-model epithelial cells treated with mevastatin show a decrease in plasma membrane cholesterol content similar to what is seen with resveratrol treatment (0.07 pA +/- 0.01, p-value <0.001) (Figure 8C, D). In the presence of AMPK inhibition (AMPKi compound C, 10 μm for 24 h), the resveratrol-mediated drop in membrane cholesterol is blocked (1.07 +/- 0.09 fold over RSV treated 9/HTEo- pCEPR (CF) epithelial cells, p-value <0.001) (Figure 8E, F).

### In vivo modulation of membrane cholesterol content by resveratrol in CF mice

**Figure 9:**
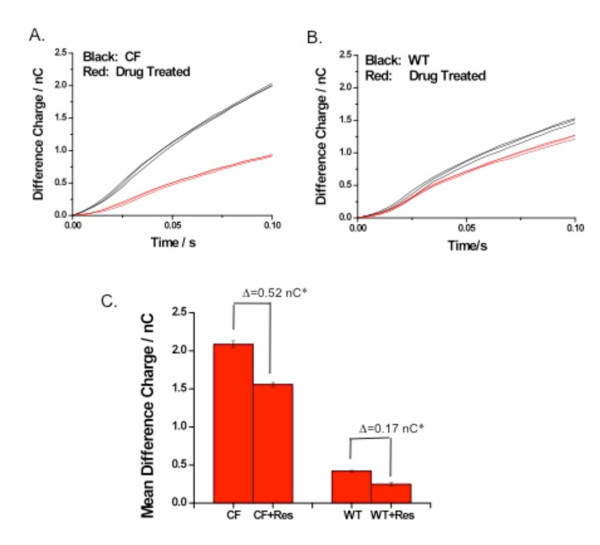
***In vivo *modulation of cholesterol in CF and wt mice with resveratrol**. Membrane cholesterol measurement in *Cftr *-/- mouse (A) nasal epithelium and in *Cftr *+/+ mouse (B) nasal epithelium in response to *in vivo *exposure to resveratrol (drug treated, RES) (15-28 mg/kg body weight) for 3 to 6 weeks. Representative tracings of electrochemical detection of membrane cholesterol are shown. Shown are three separate tracings (separate electrodes) from tissue from a single mouse. C) Tissues from three treated and three untreated *Cftr *-/- mice were examined by eight separate electrodes and means represented in bar graph. Tissues from three treated and three untreated *Cftr *+/+ mice were examined by six separate electrodes and means represented in bar graph *Significance determined from n = 3, p-value < 0.05 as determined by *t*-test.

The cell lines used for the studies above were chosen since they are known to exhibit CF-related alterations to cholesterol accumulation and membrane cholesterol [6]. However, they are a clonal cell line that may not completely reflect physiological responses. To determine whether the same intervention would have a similar impact on membrane cholesterol in a more physiologically relevant model, CFTR deficient (*CFTR*^*tm1Unc*^) mice and wt controls were treated with resveratrol for 3-6 weeks. Resveratrol was placed in the drinking water as previously described [21] and the average amount of resveratrol consumed was 15-28 mg/kg/day per mouse. The drug was tolerated well by all treatment groups. Resveratrol was chosen over mevastatin for ease of administration over an extended period of time and since it has been shown to be effective *in vivo *in mice previously [21]. All mice had adequate weight gain and lived until the time of sacrifice. After sacrifice, membrane cholesterol accessibility was determined from excised nasal epithelial in wt and *CFTR*^*tm1Unc *^mice (*Cftr -/-*) in the presence and absence of resveratrol. CF mice exhibited a decrease in membrane cholesterol detection in response to resveratrol compared to sibling untreated mice (Figure 9) (CF treated: 2.08 +/- 0.05 nC v. CF control: 1.56 +/- 0.03 nC), p-value 0.05, n = 3). Membrane cholesterol detection also decreased in wt mice treated with resveratrol (wt treated: 0.25 +/- 0.02 nC v. wt control: 0.42 +/- 0.01 nC), p-value 0.05, n = 3). The magnitude of response to resveratrol in CF mice (0.52 nC) is greater than observed in wt (0.17 nC), likely due to the increased signal in CF tissues and increased cholesterol synthesis. These data support the above cellular studies and demonstrate that cholesterol homeostasis is responsive to resveratrol *in vivo *and that electrochemical detection of membrane cholesterol is capable of monitoring responses.

### Cholesterol synthesis

**Figure 10:**
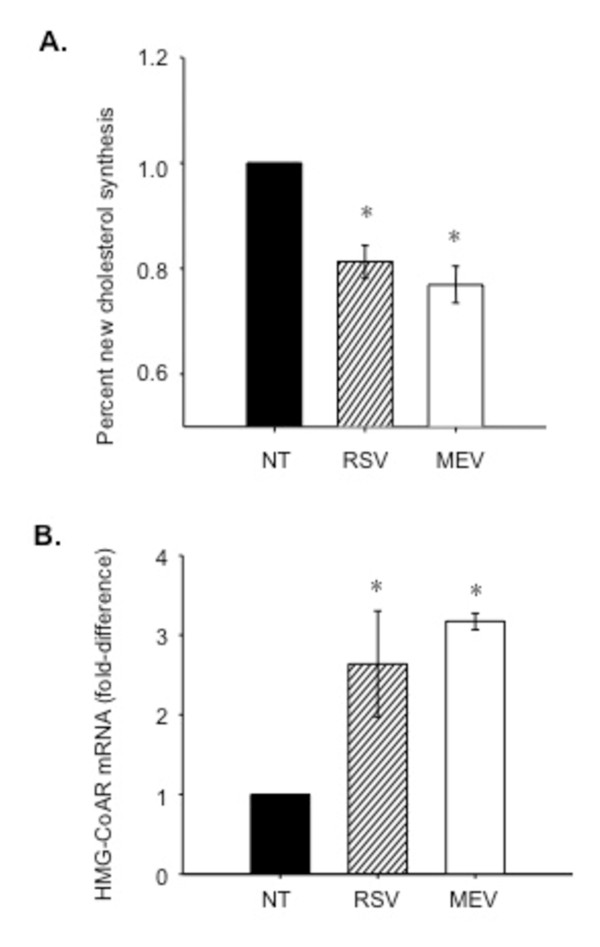
**Cholesterol synthesis and HMG-CoAR mRNA expression in CF epithelial cells**. (A) Cholesterol synthesis decreases by 20% in resveratrol treated 9HTEo-pCEPR (CF-model) epithelial cells compared to vehicle (DMSO) treated cells (0.8 +/- 0.03 fold below untreated cells *p-value <0.01). Mevastatin treated CF epithelial cells show a similar decrease in cholesterol synthesis compared to vehicle treated CF epithelial cells (0.78 +/- 0.05, *p-value 0.04). Data are the mean of three separate experiments. (B) HMG-CoAR mRNA expression in resveratrol (RSV) treated 9HTEo-pCEPR CF-model epithelial cells. An increase in HMG-CoAR mRNA levels is seen with resveratrol (50 μm, 24 h) treatment (2.6 +/- 0.6 fold over untreated cells, *p-value 0.03). Mevastatin (MEV) used as a positive control shows a similar increase in HMG-CoAR expression (3.172 +/- 0.1 fold over untreated cells, *p-value 0.04). Data are the mean of three separate experiments. Error bars are SEM. Mann-Whitney rank sum test used where normality test failed.

To verify that mevastatin and resveratrol do inhibit cholesterol synthesis, cholesterol synthesis was measured in CF-model 9/HTEo-pCEPR epithelial cells by deuterium labelling. Resveratrol treated CF cells exhibit a 20 percent decrease in cholesterol synthesis compared to vehicle treated cells (0.80 +/- 0.03 fold below untreated cells, p-value 0.005). Mevastatin, as a positive control, demonstrates a similar decrease in cholesterol synthesis in CF epithelial cells (Figure 10A).

### HMG-CoAR expression in resveratrol treated CF epithelial cells

To further verify the direct measures of cholesterol synthesis above, an indirect measure of biochemical response to reduced cholesterol synthesis was examined. A response to decreased cholesterol synthesis is up-regulation of the sterol response element (SRE) pathway to increase the expression of synthesis enzymes such as HMG-CoAR. HMG-CoAR mRNA levels were examined by RT-PCR in response to resveratrol in CF model 9HTEo-pCEPR epithelial cells. A 2.6 fold increase in HMG-CoAR mRNA levels is seen in resveratrol treated CF cells (2.6 +/- 0.6 fold over untreated cells, p-value 0.025) compared to untreated CF cells (Figure 10B). This finding is consistent with findings of cholesterol synthesis inhibition by resveratrol. A similar increase in HMG-CoAR expression is seen in mevastatin treated cells as a positive control. These results support the findings that resveratrol inhibits cholesterol synthesis

## Discussion

Membrane cholesterol is capable of modulating a variety of cellular functions including the regulation of ion channels such as ENaC and the formation of signaling complexes [22]. In addition to intracellular perinuclear accumulation of free cholesterol, our previous work has identified a CF-related increase in membrane cholesterol accessible to electrochemical detection and in increase in *de novo *cholesterol synthesis [3]. Excess cholesterol can be stored in membrane fractions and initially it was thought that excess membrane cholesterol in CF cells was the result of passive diffusion of excess cholesterol to the membrane. Given the potential importance of chronic alterations in membrane cholesterol and *de novo *synthesis, the goal of this manuscript is to determine if CFTR genotype influences these outcome measures. Regardless if cholesterol-processing alterations prove to be critical to CF pathogenesis, cholesterol measurements could prove to be important biomarkers for CF-related cellular processes that are important to pathogenesis.

Presented data demonstrate that membrane cholesterol is responsive to CFTR genotype. R/R mice exhibit a 1.6-fold increase in membrane cholesterol content in nasal epithelium compared to sibling wt mice, whereas, ΔF/ΔF mice exhibit a 2.1-fold increase. Interestingly, acute 24 h CFTR inhibition with either CFTR_inh_-172 or Gly H101 results in a decrease in membrane cholesterol content. However, 72 h exposure to either CFTR inhibitor (replenished every 24 h) results in a rebound in cholesterol content suggesting a secondary cellular response to lost CFTR function is influencing membrane cholesterol regulation. The rebound effect does not lead to elevated membrane cholesterol as seen in CF cells and tissues [3], indicating that the conditions tested here are not completely replicating the CF situation. The lack of excess cholesterol in response to acute CFTR inhibition is unexplained. One possible explanation for observed results is that 72 h pharmacological inhibition of CFTR does not lead to sufficient free cholesterol accumulation to be distributed to the plasma membrane.

Mechanistically, *de novo *cholesterol synthesis is at least a contributing factor to membrane cholesterol. Direct measurement of cholesterol synthesis reveals that lungs from R/R mice make significantly less cholesterol than ΔF/ΔF mouse lungs, although cholesterol synthesis in both genotypes is elevated compared to respective wt controls. This ratio is consistent with relative changes in membrane cholesterol content. Also, two inhibitors of cholesterol synthesis, mevastatin and resveratrol, reduce membrane cholesterol content in CF cells. *In vivo *treatment with resveratrol also reduces nasal membrane cholesterol in *Cftr *-/- mice.

The increased cholesterol synthesis we have observed in CF cells and tissues is a likely contributor to increased membrane cholesterol content in CF, but not likely the sole contributor. The model proposed by Lange and Steck [20] fits the rebound effect we see with the two CFTR inhibitors. This model also fits our data as to why the 72 h cholesterol content does not exceed baseline levels as increased synthesis would be expected to only normalize membrane content according the to the Lange and Steck model. If cholesterol synthesis in response to impeded cholesterol transport to the plasma membrane is insufficient to drive the excess plasma membrane cholesterol seen in CF, then what is responsible? As a model we propose that mutations in CFTR disrupt cholesterol movement to the plasma membrane likely through disruption of endosomal trafficking, either directly or indirectly, which is consistent with cholesterol accumulation reported in CF cells [1-4]. This disruption leads to a chronic increase in *de novo *cholesterol synthesis as predicted by the Lange and Steck model. Finally, as free cholesterol accumulates in endosomes/lysosomes of CF cells, excess cholesterol is stored in the plasma membrane leading to excess plasma membrane cholesterol observed in CF cells and tissues. This excess storage is likely not achieved in 72 h CFTR inhibitor treatment accounting for the lack of CF-like membrane cholesterol increases.

## Conclusions

The conclusion of this study is that the measurement of membrane cholesterol accessibility to electrochemical detection is influenced by CFTR. CFTR genotype correlations with the measurement, reversion to wt levels in CF cells with the introduction of wt CFTR, and pharmacological manipulation of CFTR with two separate inhibitors influencing membrane cholesterol all point to a key role of CFTR in modulating this phenotype. It is also concluded that de novo cholesterol synthesis contributes significantly to the regulation of membrane cholesterol. The initiating step in CF-related alterations of cholesterol homeostasis appears to be loss of a CFTR-mediated movement of cholesterol to the plasma membrane followed by a subsequent increase in *de novo *cholesterol synthesis. The mechanism by which CFTR influences movement of cholesterol to the plasma membrane is unknown and under investigation. A candidate mechanism being explored is CFTR function may influence endosomal trafficking. Multiple reports demonstrate CFTR presence in the endocytic pathway, but any relationship to lipid trafficking is speculative [23-25]. Our previous data suggest that a localized increase in cAMP signaling is a strong candidate as a regulator of this system in CF [1]. Also unknown is the driving force for the ultimate increase in membrane cholesterol accessibility. One possibility is an increase in cholesterol efflux. We have measured total cholesterol in *Cftr *Δ*F/*Δ*F *mouse livers and in the CF-model 9/HTEo-pCEPR cells and neither exhibits a significant increase in total cholesterol compared to respective controls (not shown). These data may indicate that CF cells have elevated cholesterol efflux that could potentially be represented in the electrochemical detection data as discussed above. Our previous work has demonstrated an increase of expression of the cholesterol transport protein NPC1 in CF cells [4]. One of the roles of NPC1 is the transport of cholesterol to the plasma membrane from endosomes [26]. The role of increased NPC1 expression in CF cells perhaps in response to cholesterol accumulation is also being explored as a potential mechanism leading to increased cholesterol accessibility at the plasma membrane.

## Competing interests

The authors declare that they have no competing interests.

## Authors' contributions

DF, MM, RW, and DJ all contributed to electrochemical measurements of cholesterol dividing work between different CFTR inhibitors, mevastatin and resveratrol treatments, and cell and nasal tissue studies. JB designed all electrochemical measurement studies and interpreted those data. NS provided CFTR inhibitors and aided in designing those studies. JR performed studies with resveratrol and mevastatin. TK designed the study in whole, interpreted data, and wrote the manuscript.
